# Nutritional Intake, Hospital Readmissions and Length of Stay in Hospitalised Oncology Patients

**DOI:** 10.3390/cancers15051488

**Published:** 2023-02-27

**Authors:** Cecelia MacFarling Meure, Belinda Steer, Judi Porter

**Affiliations:** 1 Institute for Physical Activity and Nutrition, School of Exercise and Nutrition Sciences, Deakin University, Geelong, VIC 3220, Australia; 2 Nutrition and Speech Pathology Department, Peter MacCallum Cancer Centre, Melbourne, VIC 3000, Australia

**Keywords:** nutrition intake, length of stay, hospital readmissions

## Abstract

**Simple Summary:**

Adequate nutrition in a hospital setting is essential for achieving optimal health outcomes in oncology patients. However, the relationship between nutritional intake and clinical outcomes such as length of stay and readmission rates remains unclear. This retrospective analysis investigated interrelationships between nutritional intake and clinical outcomes in hospitalised adult oncology patients. No relationship between nutritional intake and clinical outcomes was identified. Increased malnutrition risk at admission was associated with a longer length of stay.

**Abstract:**

Background: Poor food intake is an independent risk factor for malnutrition in oncology patients, and achieving adequate nutrition is essential for optimal clinical and health outcomes. This study investigated interrelationships between nutritional intake and clinical outcomes in hospitalised adult oncology patients. Methods: Estimated nutrition intake data were obtained from patients admitted to a 117-bed tertiary cancer centre during May–July 2022. Clinical healthcare data, including length of stay (LOS) and 30-day hospital readmissions, were obtained from patient medical records. Statistical analysis, including multivariable regression analysis, assessed whether poor nutritional intake was predictive of LOS and readmissions. Results: No relationships between nutritional intake and clinical outcomes were evident. Patients at risk of malnutrition had lower mean daily energy (−898.9 kJ, *p* = 0.001) and protein (−10.34 g, *p* = 0.015) intakes. Increased malnutrition risk at admission prolonged LOS (1.33 days, *p* = 0.008). Hospital readmission rates were 20.2%, and associated with age (r = −0.133, *p* = 0.015), presence of metastases (r = 0.125, *p* = 0.02) and longer LOS (1.34 days, r = 0.145, *p* = 0.02). Sarcoma (43.5%), gynaecological (36.8%) and lung (40.0%) cancers had the highest readmission rates. Conclusions: Despite research showing the benefits of nutritional intake during hospitalisation, evidence continues to emerge on the relationship between nutritional intake and LOS and readmissions that may be confounded by malnutrition risk and cancer diagnosis.

## 1. Introduction

Cancer is a leading cause of illness and death globally, with more than 19.3 million new diagnoses and almost 10 million deaths reported in 2020 [1]. In Australia, approximately 162,000 new cancer diagnoses and 50,000 deaths were reported in 2022 [2]. As a consequence, cancer accounts for about 18% of the burden of ill health and 8.8% of health system expenditure in Australia [2].

Achieving adequate nutrition in a hospital setting is essential for optimal health outcomes in oncology patients. Unfortunately, oncology patients may fail to meet their nutritional requirements due to the side effects associated with disease and treatment [3]. The consequences of failing to identify and manage inadequate nutritional intake has clinical, environmental and economic implications. Clinical outcomes may be negatively impacted through an increased risk of malnutrition and clinical complications (including pressure injuries, prolonged recovery, increased mortality and morbidity) [4,5]. Failure to account for reduced dietary intake results in increased food waste within hospital food service systems [6]. These clinical and food service issues contribute to an overall increased financial burden within the healthcare system.

Length of hospital stay (LOS) and 30-day hospital readmission rates are both key indicators of the efficiency and effectiveness of hospital care and are significant factors for hospital management [7,8], as significant health care costs are associated with increased LOS and readmissions [7,9]. The associations between nutritional intake (specifically, energy and protein intake) and these outcomes in hospitalised oncology patient populations are not well understood, nor quantified in the literature, and may be influenced predominantly by standard oncology treatment practices and adherence to clinical guidelines for nutritional care [10]. Additionally, the importance of nutritional intake, its impact on malnutrition risk and any subsequent clinical outcomes may vary depending on cancer diagnosis/type and disease severity.

A recent review identified eleven studies that had investigated the association between nutritional intake, length of stay and hospital readmissions in oncology-specific or mixed-diagnostic populations that included oncology patients [11]. No clear patterns emerged from these relationships, likely due to the heterogeneous patient cohorts (for example mixed diagnoses within cohort) and study designs (for example, study duration, setting or intervention type and also due to the confounding effects of cancer treatment and planned versus unplanned readmissions. As such, this study aimed to investigate the interrelationships between nutritional (energy and protein) intake, length of hospital stay and 30-day readmission rates in a cohort of hospitalised adult oncology patients admitted to a specialist tertiary cancer centre.

## 2. Materials and Methods

### 2.1. Study Setting

This study was undertaken at the Peter MacCallum Cancer Centre (Peter Mac), a 117- bed specialist oncology hospital in Melbourne, Australia. This retrospective cross-sectional study was approved with a waiver of consent by the Peter MacCallum Cancer Centre Ethics Committee (HREC/86641/PMCC, 29 July 2022) and the Deakin University Human Research Ethics Committee (2022-259).

At the time of this study, nutritional care was provided by 3.8 full time equivalent dietitians, and food was prepared onsite using fresh cooking and preparation methods. The hospital used the CBORD (RS Concerto^®^, CBORD, Ithaca, NY, USA) menu management system with an embedded validated food intake tool (Mobile Intake^®^). Mobile Intake^®^ uses a 5-point visual scale to estimate the patient intake of meal components that is completed at the end of mealtime by a trained healthcare professional, and nutrient analyses is completed using the AUSNUT 2011–2013 nutrient database. The output for each individual patient includes intake data for meals (breakfast, lunch and dinner) and nutrients (protein in grams and energy in kilojoules).

### 2.2. Data Collection and Study Population

Nutritional intake data were obtained from a cross-sectional sample of patients admitted during the period 25 May to 25 July 2022. Participant eligibility included adult patients (≥18 years) who were admitted and discharged during the study period. Patients consuming regular ward meals, clear fluid or liquid and pureed foods were included. Exclusion criteria included the following: deceased during study period, nil by mouth, receiving only enteral or parental nutrition, and receiving end of life care.

Relevant clinical healthcare data was obtained from patient medical records, including cancer diagnosis (primary tumour), presence of metastasis (yes/no/unknown), age (years), sex (male/female), and patient anthropometric data, including height (cm) and weight (kg). Body mass index (BMI) was calculated for the patients from individual height and weight data according to WHO guidelines. Patient malnutrition screening tool responses (Q1a, 1b, 2) and total scores (MST 0–5) [12] were collected. ESPEN guidelines [4] were used to determine individual energy (125 kJ/kg body weight/day) and protein (1.2 g/kg body weight/day) requirements for each patient and compared to Mobile Intake^®^ estimates. The percentage of patients meeting their estimated daily energy and protein requirements was calculated. LOS and 30-day hospital readmissions data were obtained for participants post-discharge from the electronic medical records.

### 2.3. Modelling of Missing Dinner Data

Initial research plans included patient admissions across 24 h periods where dietary data were recorded for all three meals. Data would be cleaned to remove entire days when there were any occurrences of missing entire-meal Mobile Intake^®^ data. Nutritional intake would then be averaged across the patient admission to minimise significant variations in intake across multiple days of admission. Unfortunately, COVID-19 negatively affected staffing resources at the hospital site, and the dataset was incomplete for the dinner service during the study period. Dinner data was available for a subgroup of patients and was used to determine the mean distribution of energy and protein intakes across a 24 h period. A scaling factor was calculated using subgroup data and applied to the rest of the cohort to calculate mean dinner and daily intakes. The scaling factor was calculated as follows. First, average daily energy and protein intakes were calculated for each participant in the subgroup using the Mobile Intake^®^ data. Mean dinner energy and protein intakes were then calculated for the whole subgroup. The scaling factor was applied to the rest of the cohort to estimate mean dinner and mean daily energy and protein intakes. The validity of the scaling factor was assessed by using the scaling factor to model dinner intake for the subgroup patients and compare the results to the Mobile Intake^®^ estimates. Paired t-tests and Mann–Whitney tests found no statistically significant differences between the modelled and Mobile Intake^®^ dinner values. 

### 2.4. Statistical Analysis

The statistical software program SPSS^®^ (IBM^®^ SPSS Statistics v.28) was used for the data analysis. A statistical significance of *p* < 0.05 was applied to all statistical analyses. Shapiro–Wilk tests were used to determine the distributions of continuous variables. The distribution of LOS was non-normally distributed with a strong positive skew and high kurtosis, so log transformation was utilised. Independent t-tests and Mann–Whitney tests were used to examine differences in continuous variables (LOS and nutritional intake) when grouped for sex, malnutrition risk at admission, metastasis, diet type and hospital readmission. Chi-squared tests were performed to investigate associations between hospital readmissions and other categorical variables (including cancer diagnoses, BMI categories, malnutrition risk and dichotomised estimated daily intake data). Kruskal–Wallis H tests were used to assess the associations between nutritional intake or LOS and type of cancer diagnosis or BMI categories. Post-hoc tests were used for pair-wise comparisons between groups. Variables considered risk factors for LOS and hospital readmissions identified in the peer reviewed literature, along with those found by this analysis to have significant associations, were entered into regression models (linear, multiple and binary logistic) to analyse associations between demographics, nutritional risk and health outcome variables. Assumptions were tested, including for collinearity, to ensure no violations and to account for confounders. 

## 3. Results

### 3.1. Participant Population Characteristics

**Figure 1:**
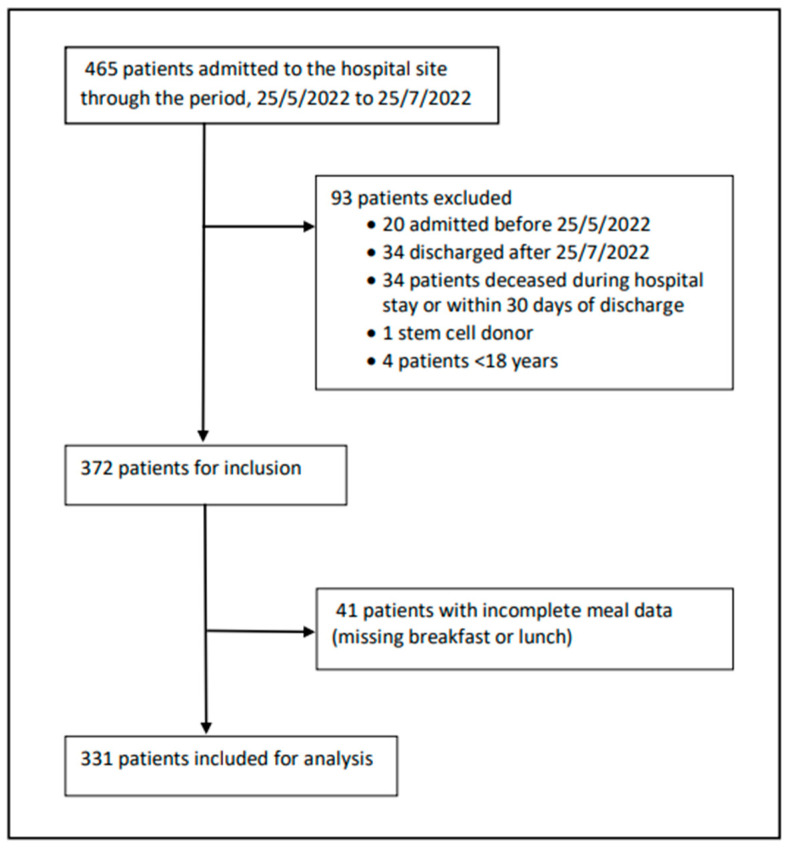
Flow chart of participant inclusion.

During the study period, food intake data were obtained post-mealtimes from 465 participants, and 71.2% (*n* = 331) were subsequently found to meet the inclusion criteria (Figure 1). 

Population characteristics at baseline are shown in Table 1. The data showed that 28.3% of participants were identified as being at risk of malnutrition at admission (MST ≥ 2). Identified malnutrition risk at admission (MST ≥ 2) was found to vary with the type of primary cancer, and the highest rates of malnutrition risk occurred in lung (48.00%), head and neck (47.62%), gynaecological (47.37%), upper gastrointestinal (40.00%) and lower gastrointestinal (35.85%) oncology participants.

### 3.2. Nutritional Intake Data

Mean daily intake was 75.22 ± 34.33 kJ/kg/d energy and 1.00 ± 0.54 g/kg/d protein. Only 9.1% of participants (*n* = 30) met their estimated daily intake for energy, and 25.4% participants (*n* = 84) met their estimated daily intake for protein through consumption of the main meals (Table 2). The majority of participants (57.1%) achieved 50% of their estimated requirements for energy, while almost 50% of participants achieved 75% of their estimated requirement for protein. Participants identified at risk of malnutrition (MST ≥ 2) at admission consumed less energy (−898.89 kJ, *p* < 0.001) and protein (−10.37 g, *p* = 0.015) compared with participants not at risk of malnutrition risk. Participants who received meals from the standard ward menu during admission consumed higher mean daily energy (1142.80 kJ, *p* < 0.001) and protein (21.38 g, *p* < 0.001) intake than participants who consumed meals from several different diet codes during admission No statistically significant associations were noted between the nutritional intake variables and sex (intake/kg body weight), presence of metastasis, age or cancer diagnosis. 

### 3.3. Length of Hospital Stay

The median LOS for all participants was 5 days (IQR: 3 to 10 days), 23% of participants had a LOS greater than 10 days, and 9% had a LOS greater than 20 days. No statistically significant relationship between the nutritional intake/kilogram, bodyweight/day and LOS were found using linear regression analysis, energy/kilogram/day (*p* = 0.286) and protein/kilogram/day (*p* = 0.651). No statistically significant relationship between the dichotomised variable ‘meeting/not meeting estimated nutrition requirements’ and LOS was found (*p* > 0.05). However, participants assigned meals exclusively from the standard ward menu during admission had a shorter LOS (−1.99 days, *p* < 0.001) than participants who consumed meals from multiple diet codes during their admission. Participants at risk of malnutrition (MST ≥ 2) were found to have a longer LOS by 1.33 days (*p* = 0.008) than those who did not. No statistically significant relationship between presence of metastases, sex, age or BMI and LOS was found. 

**Figure 2:**
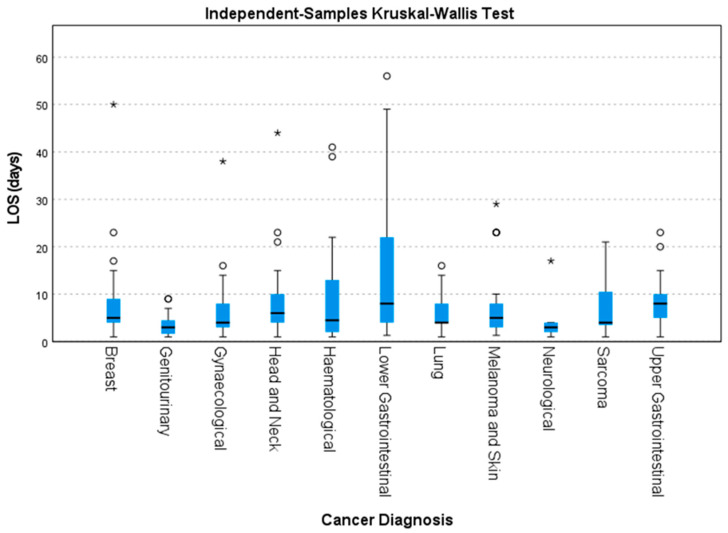
Box and whisker plot showing variability of LOS by cancer diagnosis using Kruskal–Wallis tests of independent samples. Small circles (ο) represent outliers defined as >1.5 times the IQR larger than the third quartile. Stars represent high extreme values >3 IQR above quartile 3.

Statistically significant variations in median LOS were found with differing cancer diagnoses (Figure 2) using Kruskal–Wallis H tests (*p* < 0.001). Pair-wise comparisons identified statistically significant differences in median LOS between genitourinary and lower gastrointestinal (median LOS difference of 5 days, *p* = 0.000) and genitourinary and upper gastrointestinal (median LOS difference of 5 days, *p* = 0.010) cancers.

The multiple regression model explained 10.2% of the variance in LOS (adjusted R^2^ = 0.102, *p* < 0.001). The largest contributor was inpatient diet type, and a longer LOS was associated with consuming meals from multiple diet codes compared to consuming standard ward meals (beta: 0.273, 95% CI: 0.162, 0.384, *p* < 0.001). 

### 3.4. Hospital Readmissions

**Figure 3:**
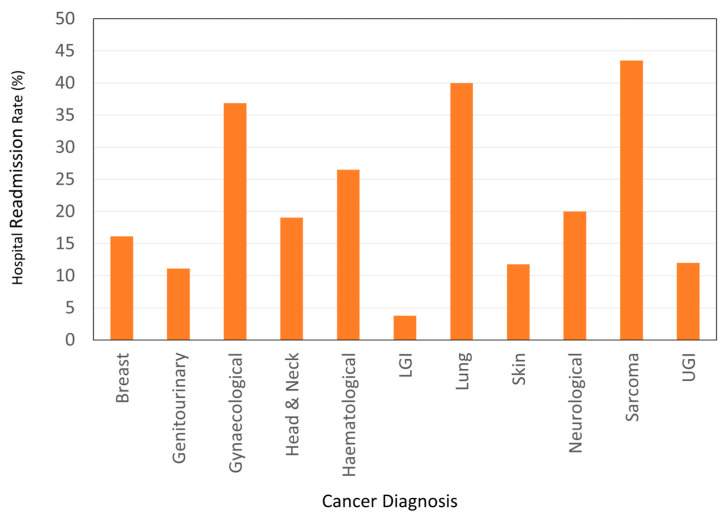
Variability of 30-day, all-cause hospital readmissions by cancer diagnosis. Note: LGI = lower gastrointestinal cancer and UGI = upper gastrointestinal cancer.

All-cause, 30-day hospital readmission rate was 20.2% (*n* = 67). No association was found between readmissions and mean daily nutritional intake per kilogram or the dichotomised variable ‘meets/does not meet estimated nutrient requirements’. Participants identified as at risk of malnutrition at admission (MST ≥ 2) had higher readmission rates (25.5%) than those who were not at risk of malnutrition (MST = 0–1) at admission (18.1%); however, this association was not found to be statistically significant using chi^2^ tests (*p* = 0.131). Age (r = −0.133, *p* = 0.015) and presence of metastasis (r = 0.125, *p* = 0.02) were found to have a small but statistically significant positive correlation with hospital readmissions. A longer LOS (1.34 days, *p* = 0.02) was also associated with fewer readmissions within 30 days. A statistically significant negative correlation between LOS and readmissions was found (r = −0.128, *p* = 0.02). Hospital readmission rates were shown to vary by cancer type (*p* < 0.001) (Figure 3). Sarcomas (43.48%) and gynaecological (36.84%) and lung (40.00%) cancers had the highest rates of hospital readmissions. 

Muliple logistic regression modelling was used to analyse the realtionship bewteen readmissions, LOS, age, malnutrition risk, metastasis and cancer diagnosis. The independent variables explained 23.9% of the variability in hospital readmissions in the model (Nagelkerke R^2^ = 0.239).

## 4. Discussion

Despite participants receiving a high standard of nutritional care in a specialist oncology setting [13], the majority did not achieve their estimated nutrient requirements for energy and protein through consumption of three main hospital meals. The inclusion of participants consuming a range of meal types during admission, including diet types that are accepted as nutritionally inadequate for short-term clinical use (i.e., clear liquid or free liquid diets), may explain some of these findings. The statistical analysis demonstrated that these participants consumed less than their standard menu counterparts and were admitted for a longer duration. Participants may move through several different diet codes during admission in response to treatment (e.g., pre-or-post surgery) or clinical side-effects (e.g., bowel obstruction, diverticulitis or Chyle leak). The inclusion of this data reflects real-world, whole-stay mean nutritional intake. Therefore, intake was associated with standard treatment plans relevant to individual cancer diagnosis. Increased requirements for energy and protein associated with cancer and poor appetite are other plausible explanations for these findings. 

No direct association between LOS and intake of energy and protein were identified. This finding is consistent with previous studies of mixed diagnostic cohorts of inpatients [14,15,16,17,18,19,20] and a recent systematic review [21]. However, an association between increased intake and a shorter LOS has been noted in several oncology-only cohorts [22,23,24]. 

LOS was associated with patient consumption of nutritionally inadequate meals during admission and identified malnutrition risk at admission (MST ≥ 2). Poor nutritional intake during hospitalisation increases malnutrition risk, which is an independent risk factor for an increased LOS [14,25,26]. This study found that participants with MST ≥ 2 had lower mean daily energy (−898.89 kJ, *p* < 0.001) and protein (−10.37 g, *p* = 0.015) intake, and MST ≥ 2 was associated with a longer LOS (1.33 days, *p* = 0.008). This association has previously been observed in several studies [14,20,23]. Studies may screen participants for malnutrition risk at inclusion [16,18,27] to focus nutrition interventions on these populations. However, participants may be discharged prior to meeting nutritional goals due to hospital treatment priorities and resource allocation [18]. Previous research suggests that a minimum daily difference in energy (155 kJ/kg) and protein (1.4 g/kg) consumption may be required to impact LOS in intervention trials [28,29].

No association between 30-day, all-cause hospital readmissions and intake of energy and protein was identified in the present study. Readmissions were associated with LOS, age and cancer diagnosis (particularly sarcomas, gynaecological and lung cancers). These findings are consistent with several previous studies [14,18,19,27,30] but not with a recent meta-analysis [21]. Heterogeneity of study designs, for example, all-cause readmissions versus unplanned readmissions and length of time between discharge and readmission (30 vs. 90 days), as well as population differences, for example, mixed-diagnosis vs oncology inpatients, may preclude generalisations. 

Poor nutritional intake during hospitalisation has been identified as a risk factor for malnutrition and increased readmission rates. This study noted that 30-day, all-cause hospital readmission rates were higher in participants with MST ≥ 2 at admission (25.5% vs. 18.1%), although it did not reach statistical significance. Calleja-Fernandez et al. found that 47% of haematology-oncology patients were at risk of malnutrition, and this elevated malnutrition risk increased 30-day hospital readmissions from 8% to 31% [19]. A recent meta-analysis found increased mean intake of energy (365 kcal; 95% CI, 272–458 kcal) and protein (17.7 g; 95% CI, 12.1–23.3 g) decreased hospital readmissions from 18.0% to 14.7% [21] in patients at nutritional risk. 

The readmission data assessed reflects all-cause readmissions rather than unplanned readmissions. It is noted that some patients’ admissions were part of standard care, as some cancer diagnoses (e.g., sarcomas) require inpatient chemotherapy. Relative percentages of unplanned versus scheduled readmissions for treatment are unknown. Readmission (whether planned or unplanned) to other hospitals was also not available, potentially underestimating readmission rates. Emergency department visits, which may reflect adverse clinical outcomes (uncontrolled pain, nausea or infection) and unplanned hospital resource use, were not captured in this analysis.

Strengths of this study include the large cohort and availability of a comprehensive array of clinical variables. The inclusion of a range of inpatient diet types reflected real-world inpatient intake; however, this introduced heterogeneity. Limitations to this study include the short duration and missing dietary intake data between meals and at dinner. However, the subset of dinner data that were available was from a patient group that was a representative sample of the overall population (age, sex, metastasis, diagnosis). The Mobile Intake^®^ system may under or over-estimate dietary intake, and the food database does not account for variability in the size and energy density of fresh foods. It is noted that the Mobile Intake^®^ provides an incomplete picture of nutritional intake, as snacks, oral nutritional support products or food brought from outside the hospital by friends and family members are not accounted for in the Mobile Intake^®^ system, and, consequently, dietary intake may be underestimated. This represents a known limitation of the data. The dietary intake reported here reflects the reality of standard hospital food and nutritional care in a specialist Australian oncology hospital and may not be generalisable to other hospital settings. Finally, it is recognised that variations in the median LOS were related to diagnosis. Additional statistical analysis using oncology subgroups could clarify the within group variations of the LOS in relation to nutritional intake; however, small subgroup numbers preclude this analysis here. 

Future research should focus on cancer diagnoses with increased malnutrition risk to optimise the impact of inpatient nutrition and the corresponding impact on clinical outcomes. A better discrimination between planned and unplanned hospital readmissions may allow for better understanding of the factors affecting unplanned readmission and allow for the early identification of at-risk patients and better allocation of hospital resource use. Additionally, the inclusion of unexpected presentations at emergency rooms may add to the current understanding of hospital resource use and patient outcomes, and collection of serum biochemistry may add to the assessment of nutritional status. Further research could also investigate the impact of nutritional intake on oncology patients with multiple comorbidities. The evaluation of quality-of-life factors in relation to nutritional intake was beyond the scope of this study; however, the impact of dietary intake on these variables would provide valuable insights to clinicians.

## 5. Conclusions

This study estimated nutritional intake data for an oncology cohort over a two-month period and analysed the interrelationships between LOS and hospital readmission rates. There was no evidence of nutritional (energy and protein) intake directly impacting the LOS or 30-day readmission rates in hospitalised adult oncology patients admitted to a tertiary cancer centre. However, increased LOS and hospital readmission rates were associated with the consumption of nutritionally inadequate meals during admission, increased malnutrition risk and cancer diagnosis.

## Figures and Tables

**Table 1:** Population characteristics.

Baseline Characteristics	Values
Age: median, IQR (years)	62 (48–62)
Sex: male, *n* (%)	188 (56.8)
Primary Diagnosis: *n* (%)	
Breast	29 (8.8)
Genitourinary	27 (8.2)
Gynaecological	19 (5.7)
Head & Neck	20 (6.0)
Haematological	68 (20.5)
Lower Gastrointestinal	53 (16.0)
Lung	25 (7.6)
Melanoma and Skin	33 (10.0)
Neurological	4 (1.2)
Sarcoma	23 (6.9)
Upper Gastrointestinal	26 (7.9)
Cancer of Unknown Primary	4 (1,2)
Presence of Metastasis: *n* (%)	142 (42.9)
Malnutrition Screening Tool ^a^ Score: *n* (%)	
0	193 (58.3)
1	44 (13.3)
2	55 (16.6)
3	31 (9.3)
4	6 (1.8)
5	2 (0.6)
Body Mass Index: kg/m^2^ (%)	
Underweight	38 (11.5)
Normal	106 (32.0)
Overweight	79 (23.9)
Obese	101 (30.5)
Unknown ^b^	7 (2.1)

^a^ Ferguson et al. [12]; ^b^ unknown BMI due to height data not collected during admission.

**Table 2:** The number and percentage of participants meeting estimated nutrient requirements.

	Energy				Protein			
Meets Estimated Nutrient Requirements ^a^	25%	50%	75%	100%	25%	50%	75%	100%
*n*	315	189	77	30	320	261	162	84
%	95.2%	57.1%	23.3%	9.1%	96.7%	78.9%	48.9%	25.4%

^a^ Calculated as estimated nutritional intake compared against ESPEN guidelines [4].

## Data Availability

Ethics approvals limit the sharing of participant level data.
